# Supplementary material for: Heterogeneity and ontogeny of mouse thymic macrophages reveal a requirement for Csf1r-expressing myeloid cells during early T cell development
Source: eLife. 2026 Oct 7;15:RP109219. doi: 10.7554/eLife.109219 (PMC13645347; doi:10.7554/eLife.109219)
Supplement: Supplementary file 1 [file elife-109219-mdarchecklist1.docx]

**Materials Design Analysis Reporting (MDAR)**

**Materials:**

| **Newly created materials** | Not applicable; no newly created materials are reported in this study. | X |
| --- | --- | --- |
| The manuscript includes a dedicated "materials availability statement" providing transparent disclosure about availability of newly created materials including details on how materials can be accessed and describing any restrictions on access. | No new reagents, constructs, cell lines, or organisms are described as “this paper.” | X |
|  |  |  |
| **Antibodies** | Materials and Methods: Intravenous CD45 staining; Flow cytometry; Cell sorting; Supplemental Tables 1–2; Key Resources Table. | **N/A** |
| For commercial reagents, provide supplier name, catalogue number and [RRID](https://scicrunch.org/resources), if available. | Commercial antibodies are reported with supplier, clone, fluorochrome, dilution, catalog number and RRID in the Key Resources Table; use context is given in Methods and Supplemental Tables 1–2. |  |
|  |  |  |
| **DNA and RNA sequences** | Not applicable; no novel DNA/RNA sequences, primers, probes, or oligonucleotides are reported. | X |
| Short novel DNA or RNA including primers, probes: Sequences should be included or deposited in a public repository. | No new sequence-based reagents are described in the manuscript. | X |
|  |  |  |
| **Cell materials** | Not applicable; no cell lines or primary cell cultures are used. | X |
| Cell lines: Provide species information, strain. Provide accession number in repository OR supplier name, catalog number, clone number, OR RRID. | No cell lines are reported. | X |
| Primary cultures: Provide species, strain, sex of origin, genetic modification status. | No primary cultures are reported; ex vivo fetal thymus organ culture uses intact mouse thymic lobes. | X |
|  |  |  |
| **Experimental animals** | Materials and Methods: Animals; Timed pregnancies and embryonic thymus analysis; Figure legends; Key Resources Table. | **N/A** |
| Laboratory animals or Model organisms: Provide species, strain, sex, age, genetic modification status. Provide accession number in repository OR supplier name, catalog number, clone number, OR RRID. | Mouse species/strains, ages, and genetic modification status are reported in Animals and Timed pregnancies and summarized in the KRT with JAX/RRID identifiers. Sex is reported for breeder males and females in timed matings, but not uniformly for all experimental cohorts. |  |
| Animal observed in or captured from the field: Provide species, sex, and age where possible. | No field-captured animals were used. | X |
|  |  |  |
| **Plants and microbes** | Not applicable; no plants or microbes were used. | X |
| Plants: provide species and strain, ecotype and cultivar where relevant, unique accession number if available, and source (including location for collected wild specimens). | No plants were used. | X |
| Microbes: provide species and strain, unique accession number if available, and source. | No microbes were used. | X |
|  |  |  |
| **Human research participants** | Not applicable; no human research participants were involved. | X |
| If collected and within the bounds of privacy constraints report on age, sex, gender and ethnicity for all study participants. | No human participant demographics apply to this study. | X |

**Design:**

| **Study protocol** | Not applicable; the study was not pre-registered and is not a clinical trial. | X |
| --- | --- | --- |
| If the study protocol has been pre-registered, provide DOI. For clinical trials, provide the trial registration number OR cite DOI. | No study protocol DOI/trial registration is reported. | X |
|  |  |  |
| **Laboratory protocol** | Materials and Methods: Cell isolation from thymus; Cell sorting; Fetal thymus organ culture (FTOC) and TM depletion in MaFIA mice; Analysis of scRNA-Seq and data integration; Trajectory analysis using Monocle3. | **N/A** |
| Provide DOI OR other citation details if detailed step-by-step protocols are available. | Detailed methods are described in the manuscript. Manufacturer instructions are cited for Miltenyi dissociation / gentleMACS and 10x library preparation; FTOC is referenced to Han et al. 2021 and Nitta et al. 2013 (refs 29,30). |  |
|  |  |  |
| **Experimental study design (statistics details) *** | | |
| **For in vivo studies: State whether and how the following have been done** | **Indicate where provided: section/figure legend. If it could have been done, but was not, write “not done”** | **N/A** |
| Sample size determination | Materials and Methods: Study design / Statistical analysis. Sample sizes were selected on the basis of prior experience with these mouse models, fetal thymic organ culture assays, and thymic macrophage flow cytometric analyses; no formal a priori power calculation was performed. |  |
| Randomisation | Materials and Methods: Study design; Fetal thymus organ culture (FTOC) and TM depletion in MaFIA mice. For ex vivo FTOC experiments, littermate thymic lobes were allocated to treatment groups at the time of culture setup so that control and AP20187-treated samples were represented within each experiment; formal randomisation was not used. |  |
| Blinding | Materials and Methods: Study design / Statistical analysis. Investigators were not blinded during sample allocation, but flow cytometry gating and downstream quantitative analyses were performed using prespecified marker combinations and analysis pipelines applied uniformly across all groups. |  |
| Inclusion/exclusion criteria | Materials and Methods: Study design; Transcriptomic analysis; Analysis of scRNA-Seq and data integration. Samples were excluded only for predefined technical quality-control reasons, including poor cell recovery, failed staining/acquisition, or failure to meet the scRNA-seq filtering thresholds described in Methods; exclusions were technical rather than outcome-based. |  |
|  |  |  |
| **Sample definition and in-laboratory replication** | Figure legends; Materials and Methods: Cell sorting; Transcriptomic analysis. | **N/A** |
| State number of times the experiment was replicated in the laboratory. | Figure legends; Materials and Methods: Cell sorting; Transcriptomic analysis; Study design. Replication is reported in figure legends as n per group and number of independent experiments; scRNA-seq was performed in two independent experiments yielding 3,214 and 2,815 cells. |  |
| Define whether data describe technical or biological replicates. | Materials and Methods: Study design; figure legends. Each mouse or independently established fetal thymic organ culture was treated as one biological replicate; scRNA-seq cells are technical observations nested within two independent biological experiments. |  |
|  |  |  |
| **Ethics** | Materials and Methods: Animals; submission form. | **N/A** |
| Studies involving human participants: State details of authority granting ethics approval (IRB or equivalent committee(s), provide reference number for approval. | Not applicable; no human participants were involved. | X |
| Studies involving experimental animals: State details of authority granting ethics approval (IRB or equivalent committee(s), provide reference number for approval. | All animal procedures were approved by the Sunnybrook Health Sciences Centre Animal Care Committee (Toronto, Ontario, Canada) under AUP 2024-0138. |  |
| Studies involving specimen and field samples: State if relevant permits obtained, provide details of authority approving study; if none were required, explain why. | Not applicable; no specimen or field samples were collected. | X |
|  |  |  |
| **Dual Use Research of Concern (DURC)** | Not applicable; this study does not appear to fall under DURC regulations. | X |
| If study is subject to dual use research of concern regulations, state the authority granting approval and reference number for the regulatory approval. | No DURC approval is required/reported. | X |

**Analysis:**

| **Attrition** | Materials and Methods: Study design; Transcriptomic analysis; Analysis of scRNA-Seq and data integration. | **N/A** |
| --- | --- | --- |
| Describe whether exclusion criteria were pre-established. Report if sample or data points were omitted from analysis. If yes, report if this was due to attrition or intentional exclusion and provide justification. | Exclusion criteria were predefined at the technical level. No animals or samples were excluded unless they failed technical quality-control criteria, including poor cell recovery, failed staining/acquisition, or failure to meet the scRNA-seq filtering thresholds described in Methods; all exclusions were technical rather than outcome-based. |  |
|  |  |  |
| **Statistics** | Materials and Methods: Statistical analysis; relevant figure legends. | **N/A** |
| Describe statistical tests used and justify choice of tests. | Statistical tests are described in Statistical analysis: Mann-Whitney U, Kruskal-Wallis with Dunn's post hoc test, Holm-Šídák multiple-comparison correction, D'Agostino-Pearson normality testing, Brown-Forsythe and residual-plot assessment of variance assumptions; data are presented as mean ± SEM. |  |
|  |  |  |
| **Data availability** | Data Availability statement; Materials and Methods: Analysis of scRNA-Seq and data integration. | **N/A** |
| For newly created and reused datasets, the manuscript includes a data availability statement that provides details for access (or notes restrictions on access). | A dedicated Data Availability statement indicates that newly generated scRNA-seq data are deposited in GEO under accession GSE32659 and that reused public datasets are available under GEO accessions GSE185460 and GSE188647. |  |
| When newly created datasets are publicly available, provide accession number in repository OR DOI and licensing details where available. | Newly generated scRNA-seq data are publicly available in GEO under accession GSE32659. |  |
| If reused data is publicly available provide accession number in repository OR DOI, OR URL, OR citation. | Reused public datasets are identified in Methods and the Data Availability statement as GEO:GSE185460 and GEO:GSE188647. |  |
|  |  |  |
| **Code availability** | Data Availability statement; Materials and Methods: Flow cytometry; Analysis of scRNA-Seq and data integration; Trajectory analysis using Monocle3; Statistical analysis; Key Resources Table. | **N/A** |
| For any computer code/software/mathematical algorithms essential for replicating the main findings of the study, whether newly generated or re-used, the manuscript includes a data availability statement that provides details for access or notes restrictions. | The Data Availability statement notes that no newly generated custom code essential for reproducing the main findings was deposited; analyses were performed with publicly available software reported in Methods and the KRT (FlowJo, R 4.4.1, Seurat, Harmony, Monocle3, GraphPad Prism). |  |
| Where newly generated code is publicly available, provide accession number in repository, OR DOI OR URL and licensing details where available. State any restrictions on code availability or accessibility. | Not applicable; no newly generated custom code repository/DOI/URL is reported. |  |
| If reused code is publicly available provide accession number in repository OR DOI OR URL, OR citation. | Reused code/software is cited in Methods and the KRT; public resources include R, FlowJo, Seurat, Harmony, Monocle3, and GraphPad Prism. |  |

**Reporting:**

The MDAR framework recommends adoption of discipline-specific guidelines, established and endorsed through community initiatives.

| **Adherence to community standards** | Submission includes this MDAR checklist; animal work is reported in line with eLife reporting expectations, and relevant ARRIVE-style information on animals, study design, ethics, and resources is provided in Materials and Methods, figure legends, the Key Resources Table, and this checklist. | **N/A** |
| --- | --- | --- |
| State if relevant guidelines (e.g., ICMJE, MIBBI, ARRIVE, STRANGE) have been followed, and whether a checklist (e.g., CONSORT, PRISMA, ARRIVE) is provided with the manuscript. | This MDAR checklist is provided with the manuscript. The manuscript now reports the key animal, ethics, study-design, resource-identification, and availability information requested for eLife submission. |  |
