# Supplementary material for: Heterogeneity and ontogeny of mouse thymic macrophages reveal a requirement for Csf1r-expressing myeloid cells during early T cell development
Source: eLife. 2026 Oct 7;15:RP109219. doi: 10.7554/eLife.109219 (PMC13645347; doi:10.7554/eLife.109219)
Supplement: Supplementary file 2 [file elife-109219-supp1.docx]

**SUPPLEMENTAL TABLES**

**Supplemental Table 1**. Antibodies used for macrophage flow cytometry staining.

| **MARKER** | **CLONE** | **FLUOROCHROME** | **DILUTION** | **BRAND** |
| --- | --- | --- | --- | --- |
| CD64 | X54-5/7.1 | APC | 1:200 | BioLegend |
| F4/80 | T45-2342 | BUV805 | 1:200 | BD |
| MerTK | 2B10C42 | FITC | 1:200 | BioLegend |
| TIMD4 | RMT4-54 | PE | 1:200 | BioLegend |
| VCAM1 | 429 | PECy7 | 1:200 | BioLegend |
| CX3CR1 | SA011F11 | BV605 | 1:200 | BioLegend |
| CCR2 | SA203G11 | BV785 | 1:200 | BioLegend |
| CD11b | M1/70 | APCCy7 | 1:200 | BioLegend |
| Ly6C | HK1.4 | BV711 | 1:200 | BioLegend |
| MHC-II | M5/114.15.2 | AF700 | 1:200 | BioLegend |
| CD11c | N418 | BUV395 | 1:200 | BD |
| CD45 | 30-F11 | BUV563 | 1:150 | BD |
| CD16/32 | 93 | - | 1:100 | BioLegend |

**Supplemental Table 2.** Antibodies used for T cell flow cytometry staining.

| **MARKER** | **CLONE** | **FLUOROCHROME** | **DILUTION** | **BRAND** |
| --- | --- | --- | --- | --- |
| CD3e | 145-2C11 | PECy7 | 1:200 | BioLegend |
| CD4 | RM4-5 | BV711 | 1:200 | BioLegend |
| CD8a | 53-6.7 | BUV395 | 1:200 | BD |
| CD25 | PC61 | APCCy7 | 1:200 | BioLegend |
| CD44 | IM7 | BV785 | 1:200 | BioLegend |
| B220 | RA3-6B2 | AF700 | 1:200 | BioLegend |
| CD45 | 30-F11 | BUV563 | 1:150 | BD |
| CD16/32 | 93 | - | 1:100 | BioLegend |
