# Supplementary material for: Heterogeneity and ontogeny of mouse thymic macrophages reveal a requirement for Csf1r-expressing myeloid cells during early T cell development
Source: eLife. 2026 Oct 7;15:RP109219. doi: 10.7554/eLife.109219 (PMC13645347; doi:10.7554/eLife.109219)
Supplement: Peer review history

# Editor report: eLife Assessment

**DOI:** 10.7554/eLife.109219.3.sa0

**Authors:** Sarah Russell

**Affiliations:** Peter MacCallum Cancer Centre Australia

**Keywords:** Convincing, Important

This study characterises the heterogeneity and developmental origins of macrophages in the thymus and demonstrates a role for Csf1r-expressing myeloid cells in the first step of T cell development. The state-of-the-art experiments provide **convincing** evidence, and the assessors concerns were addressed in the revised submission. These results provide an **important** advance that furthers our understanding of thymus biology, especially in view of the contribution of heterogenous thymic macrophage subpopulations.

# Referee report: Reviewer #1 (Public review)

**DOI:** 10.7554/eLife.109219.3.sa1

[Editors' note: this version has been assessed by the Reviewing Editor without further input from the original reviewers. The authors have addressed the comments raised in the previous round of review.]

Summary:

The current manuscript characterizes in detail the macrophages in the thymus. The authors identify two distinct populations of thymic macrophages and describe their surface marker expression and transcriptional signatures. They also explore their ontology and kinetics of settling and persistence in the thymus and find that the TIMD4+ macrophages are derived from embryonic progenitors and self-maintain in the thymus, while the TIMD4- macrophages are derived from monocytes. Most importantly, the authors test the functional importance of thymic macrophages for T cell development using an in vitro depletion system, from which they conclude that macrophages are important for one of the earliest selection steps in T cell development - the beta selection.

Strengths:

The authors use state-of-the-art techniques, such as multiple genetically modified mice, multi-color flow cytometry, single-cell RNA sequencing, genetic fate mapping, and fetal thymic organ culture (FTOC) combined with depletion. Their work is in good agreement with prior published studies on the subject, such as Tacke et al. (PMID: 26091486) and Zhou et al. (PMID: 36449334). In addition to reproducing prior knowledge, the authors uncover novel and unexpected facets of thymic macrophage biology, such as their SpiC independence and the fact that TIMD4- thymic macrophages depend on CCR2 (Tacke et al. have shown that the overall thymic macrophage compartment is normal in CCR2-/- mice). Most surprisingly, the authors claim that thymic macrophages control an early checkpoint in T cell development, the beta selection. This has not been reported before, as beta selection is usually considered a cell-autonomous process in thymocytes that does not require input from other cells.

# Referee report: Reviewer #2 (Public review)

**DOI:** 10.7554/eLife.109219.3.sa2

This manuscript from Zuniga-Pflucker laboratory describes that thymic macrophages are heterogeneous in flow cytometric and transcriptomic profiles, containing two major populations characterized by TIMD4 and CX3CR1 expression. These macrophage populations are both parenchymal in the thymus but are unequal in developmental ontogeny, Flt3 expression history, and CCR2 dependency. The manuscript further reports the interesting findings that the depletion of thymic macrophages impairs thymocyte development at the DN3 beta-selection checkpoint. These results provide an important advance for further understanding of thymus biology, especially in view of the contribution of heterogenous thymic macrophage subpopulations.

# Author comment: Author response

**DOI:** 10.7554/eLife.109219.3.sa3

**Authors:** Helen Wang, Vinothkumar Rajan, Anthony Wong, Slava Epelman, Juan Carlos Zúñiga-Pflücker

**Affiliations:** University of Toronto Toronto Canada; University of Toronto Toronto Canada; University of Toronto Toronto Canada; Toronto General Hospital Research Institute Toronto Canada; University of Toronto Toronto Canada

The following is the authors’ response to the original reviews.

We thank the editors and reviewers for their thoughtful evaluation and helpful recommendations. These comments helped us distinguish more clearly between two complementary advances in the study. First, the phenotypic, transcriptomic, and ontogenetic analyses refine the organization of the thymic macrophage compartment and identify CCR2 dependence of the TIMD4- VCAM1+ population. Second, the MaFIA fetal thymus organ culture (FTOC) experiments reveal that an intact *Csf1r*-expressing myeloid compartment is required for efficient progression across the DN3-to-DN4 checkpoint. The revised manuscript now presents both advances directly while matching the cellular specificity of each conclusion to the experimental system that supports it.

The central functional result is supported by a coordinated set of observations: depletion of *Csf1r* expressing cells reduces CD4^+^CD8^+^ double-positive (DP) ab-lineage thymocyte production, preserves γδ T cell output, causes reciprocal accumulation of DN3 and loss of DN4 cells, and reduces CD27 expression within the DN compartment. We now emphasize that convergence, which localizes the phenotype to the b-selection transition. At the same time, because the MaFIA system targets Csf1r-expressing myeloid cells rather than a single macrophage subset, the manuscript assigns the demonstrated requirement to the thymic myeloid compartment, this now reflected on the revised title as well. This framing preserves the biological importance of the result without attributing more cellular specificity than the experiment provides.

The major revisions include:

- A revised Title and an Abstract that leads with the principal conclusions: TM specialization, unequal developmental contributions, CCR2 dependence, and a requirement for *Csf1r* expressing myeloid cells during DN3-to-DN4 progression.

- A clearer account of what Zhou et al. established and how the present work adds VCAM1-based prospective resolution, intravascular-labeling information, SpiC analysis, CCR2 dependence, and functional fetal thymus organ culture (FTOC) data.

- A Results section that explains the convergence of DN3, DN4, CD27, DP cells, and γδ T cell measurements as part of developmental transition.

- More precise interpretation of relative marker expression, pseudotime, SpiC deficiency, Tomato/GFP double-positive events, and thymocyte-associated transcripts in the scRNA-seq results.

- A focused Discussion paragraph that acknowledges the cellular breadth of the MaFIA model while retaining the conclusion that the *Csf1r*-expressing myeloid niche supports early ab T-lineage development.

- Expanded figure legends that make the gating, reporter, heatmap, MaFIA construct, and developmental interpretations easier to follow.

The revision is based on fuller analysis and clearer presentation of the results and datasets.

**eLife Assessment**

The macrophage characterisation is interesting, although the evidence for the specific involvement of macrophages in beta-selection is incomplete, as alternative explanations have not been ruled out.

We agree that the depletion experiment resolves a requirement for the Csf1r-expressing thymic myeloid compartment rather than for macrophages alone. We have revised the manuscript with this distinction in mind. Importantly, the developmental conclusion remains strong: DN3 accumulation, DN4 loss, reduced CD27 expression, and diminished CD4^+^CD8^+^ double-positive (DP) output all point to impaired progression at the b-selection checkpoint, while preserved γδ T cell output and epithelial-cell numbers argue against nonspecific failure of the entire fetal thymus organ culture (FTOC). The revised text therefore states that Csf1r-expressing myeloid cells support this early ab T cell checkpoint, while discussing the relative contributions of TMs, monocytes, and DCs as the next level of cellular resolution.

The Title, Abstract, final Results section, Discussion, Conclusion, and Figures 8-10 Legends were revised to make the positive compartment-level conclusion explicit and consistent.

**Public Reviews:**

**Reviewer #1 (Public review):**

The thymic macrophage depletion experiments are not well controlled; DCs are also depleted, the fetal thymus has little or no medulla, and direct AP20187 toxicity to DN thymocytes in MaFIA mice has not been excluded.

The reviewer identifies the key issue of cellular attribution. We have recast the experiment according to what the MaFIA system directly tests: the functional contribution of Csf1r-expressing myeloid cells within an intact FTOC. This interpretation is supported by efficient loss of both TM populations, preservation of EpCAM+ epithelial-cell numbers, and absence of TM depletion in AP20187treated non-transgenic C57BL/6 FTOCs. We no longer use adult cortical-versus-medullary anatomy to infer that macrophages must be the sole responsible population in the fetal thymus. Instead, we emphasize the experimentally secure result that perturbing the Csf1r-expressing myeloid niche produces a selective and internally consistent defect in ab T cell development at the DN3-to-DN4 transition. The possibility of contributions from DCs, monocytes, or direct transgene expression in a thymocyte fraction is addressed once, in a focused Discussion paragraph, as the rationale for assigning the conclusion at the compartment level.

The MaFIA Results section now leads with the transgene-dependent depletion and the convergent developmental phenotype; the Discussion contains a balanced statement of cellular resolution; the Title and Legends consistently refer to Csf1r-expressing myeloid cells.

**Reviewer #2 (Public review):**

Zhou et al. previously reported similar TM heterogeneity, localization, and developmental characteristics. The manuscript should distinguish prior findings from new findings and address localization in relation to beta-selection.

We have made this distinction explicit throughout. Zhou et al. established the two-population framework, their cortical versus medullary/cortico-medullary localization, their broad embryonic versus adult hematopoietic origins, and their age-associated remodeling. Building on that foundation, the present study contributes: (i) a prospective TIMD4/VCAM1 gating strategy linked to MafB and Csf1r reporters; (ii) intravascular-labeling evidence that both VCAM1+ populations are predominantly parenchymal; (iii) transcriptomic definition of efferocytic versus antigen-presentation/interferon programs; (iv) evidence that total TM abundance is maintained independently of SpiC; (v) selective CCR2 dependence of TIMD4- VCAM1+ macrophages and thymic monocytes; and (vi) functional evidence that the Csf1r-expressing thymic myeloid compartment supports DN3-to-DN4 progression. We use the anatomical localization established by Zhou et al. as the relevant biological context and reserve our new conclusions for the endpoints measured here. Accordingly, the FTOC phenotype is assigned to the Csf1r-expressing myeloid compartment rather than specifically to cortical TIMD4+ macrophages.

The Introduction now clearly separates established knowledge from the questions addressed here, and the Results and Discussion explicitly identify the study-specific advances. Reference 27 has also been corrected to the final eLife publication.

**Recommendations for the authors:**

**Reviewer #1 (Recommendations for the authors):**

(1) Trajectory analysis

The Abstract mentions trajectory analysis, but there is no corresponding Results section.

We added a Results paragraph describing the Monocle3 analysis and integrated its interpretation with the fate-mapping and CCR2 experiments. Rooting the trajectory in *Ly6c2*+ *Ccr2*+ monocytes produces a transcriptional continuum toward macrophage states, supporting progressive acquisition of macrophage programs from a monocyte-like state. We also explain why pseudotime is complementary to, but not a substitute for, genetic lineage information: it models transcriptional relationships and therefore is not used to infer direct conversion between the two mature TM populations. This rationale allows the analysis to contribute meaningfully without asking it to resolve ontogeny on its own.

The trajectory Methods were clarified and a new Results paragraph was added after the scRNA-seq specialization analysis; the Abstract now summarizes the result at the appropriate level.

(2) Comparison with the Zhou et al. gating strategy

Compare the TIMD4/VCAM1 gating strategy with the CD64/F4/80/TIMD4 definition used by Zhou et al., including the identity of TIMD4-VCAM1- cells.

We now map the two VCAM1+ gates directly onto the established TM framework. TIMD4+ VCAM1+ cells correspond to the TIMD4+ cortical population, whereas TIMD4- VCAM1+ cells show the CX3CR1 enrichment expected of the medullary/cortico-medullary population. VCAM1 therefore adds a useful prospective discriminator within the CD64+ F4/80+ parent gate. The TIMD4- VCAM1- gate is MafB-low/negative, Csf1r-EGFP-high, and enriched for Ly6C, CCR2, and CX3CR1, supporting its designation as monocyte-enriched. We use “monocyte-enriched” because it accurately captures the dominant phenotype without implying that every event in the gate is developmentally identical.

The first Results section and Figures 1-2 Legends now explain this correspondence and terminology.

(3) Difference in IV-CD45 labeling

Why are approximately 40% of Ly6C+CD11b+ cells labeled, but only approximately 10% of TIMD4-VCAM1- cells?

The percentages arise from different denominators. Ly6C+ CD11b+ is a broad myeloid gate that contains both blood-exposed and parenchymal cells. TIMD4- VCAM1- is a narrower population defined within the CD64+ F4/80+ parent gate and therefore samples a different compartment. We now make that gating relationship explicit. The i.v.-labeling experiment consequently supports two positive conclusions: both VCAM1+ macrophage populations are predominantly parenchymal, and the broad Ly6C+ CD11b+ compartment contains a substantially larger blood-exposed component.

The i.v.-labeling Results paragraph and Figure 2 Legend now describe the two gates and their distinct denominators.

(4) SpiC requirement in individual TM populations

Determine proportions and numbers of TM subpopulations in Spic-/- mice.

The biological rationale for this suggestion is strong because Spic is enriched in TIMD4+ VCAM1+ macrophages. Figure 3C, however, quantifies the aggregate CD64+ F4/80+ TM compartment. We therefore revised the conclusion to the level directly supported by that experiment: total TM abundance is maintained in Spic-/- mice despite the expected loss of splenic red pulp macrophages. This is an informative distinction because it shows that the overall thymic macrophage compartment does not share the obligate SpiC dependence of red pulp macrophages, even though a subset-selective quantitative or functional effect remains a question for future work.

The Figure 3 Results paragraph, Discussion, and legend now state preservation of total TM abundance rather than making a population-by-population claim.

(5) Tomato/GFP double-positive cells

Many cells appear to express both GFP and tdTomato. What does this mean?

We expanded the explanation of the mTmG reporter. Flt3-Cre-mediated recombination initiates a switch from membrane Tomato to membrane GFP, but the pre-existing membrane Tomato protein need not disappear instantaneously. Tomato+ GFP+ events are therefore consistent with recent/incomplete reporter transition or persistence of stable Tomato protein after recombination. We do not treat them as a third ontogenetic lineage. The key comparative result is the distribution of reporter histories across populations: TIMD4+ VCAM1+ macrophages retain a large FLT3-independent fraction, whereas TIMD4- VCAM1+ macrophages and monocytes show substantially greater FLT3 history.

The fate-mapping Results paragraph and Figure 6 Legend now define the reporter transition and the interpretation of double-positive events.

(6) Direct AP20187 toxicity in DN thymocytes

Test AP20187 toxicity in MaFIA and control DN thymocytes, for example by active caspase-3 staining.

The proposed caspase-3 analysis is designed to determine whether a DN thymocyte fraction expresses sufficient MaFIA transgene to be directly affected. The existing controls establish two important features of the result: AP20187 does not reduce TM numbers in non-transgenic C57BL/6 FTOCs, demonstrating transgene dependence, and the developmental response is patterned rather than global, with preserved γδ T cell output, DN3 accumulation, DN4 loss, and reduced CD27 expression. These convergent observations support the conclusion that integrity of the Csf1r-expressing myeloid compartment is required for efficient DN3-to-DN4 progression. We now state that conclusion prominently and note the remaining question of thymocyte-intrinsic transgene activity once, in the focused Discussion paragraph.

The MaFIA Results now emphasize the transgene-dependent control and convergent developmental measurements; the Discussion states the remaining cellular-resolution issue in one focused paragraph.

**Reviewer #2 (Recommendations for the authors):**

(1) Figure 1 color annotation

Clarify whether the color annotation in panel C is applied to panel F.

Yes. Panel F evaluates MafB-mCherry and Csf1r-EGFP reporter expression within the same TIMD4/VCAM1-defined populations shown in panel C. We now state this explicitly so the reporter patterns can be interpreted as independent support for the identity of each prospectively defined gate. We also removed the previous reference to immunofluorescence localization because localization data are not displayed in Figure 1.

Figure 1 Legend revised.

(2) Figure 2 controls, thresholds, and population identities

Provide staining controls and thresholds; reconsider categorical “lack” statements; clarify Ly6C+CD11b+ and CD64+F4/80+ populations and the apparent IV-CD45 discrepancies.

The underlying interpretive point is whether marker expression is categorical or relative. We revised the text to describe Ly6C, CCR2, and CX3CR1 comparatively, which more faithfully represents continuous flow-cytometric measurements. This improves the biological conclusion: both VCAM1+ populations are Ly6C-low/negative and CCR2-low relative to the TIMD4- VCAM1- monocyte-enriched population, while CX3CR1 is enriched in TIMD4- VCAM1+ relative to TIMD4+ VCAM1+ macrophages. We also define the broad Ly6C+ CD11b+ comparison gate, the CD64+ F4/80+ macrophage parent gate, and the narrower TIMD4/VCAM1 subgates. With those denominators made explicit, the iv-CD45 measurements become internally consistent rather than apparently contradictory.

The Figure 2 Results paragraph and legend now use relative marker language and explain the gate hierarchy.

(3) Figure 3 colors, VCAM1, and SpiC populations

Explain panel B colors and the apparent Vcam1 difference; provide individual TM-population data in Spic-/- mice.

Panel B is a row-scaled expression heatmap: yellow and purple indicate relatively higher and lower scaled expression for each gene, respectively. These colors should not be read as absolute expression or compared directly with antibody fluorescence, because transcript abundance and cell-surface protein are regulated at different levels and have different dynamic ranges. The legend now makes this distinction explicit. Vcam1 transcript enrichment is nevertheless consistent with VCAM1 protein being a useful surface discriminator in our gating scheme. For SpiC, we now limit the conclusion to the measurement displayed in panel C, as preservation of total CD64+ F4/80+ TMs, while explaining the biological significance of their divergence from SpiC-dependent splenic red pulp macrophages.

Figure 3 Results, Discussion, and Legend revised.

(4) Figure 4 genes not shown

H-2K, H-2D, and Runx3 are described but not shown in the figure.

We clarified the division of information between Figure 4 and Table 1. Figure 4 displays pathway-level GO enrichment, which supports the higher-order conclusion that TIMD4- VCAM1+ macrophages are enriched for antigen-presentation and interferon-response programs. Individual genes contributing to the subset signatures, including H2-K1, H2-D1, and Runx3, are reported in Table 1. The revised wording no longer implies that those individual genes are plotted in Figure 4.

Figure 4 Results paragraph and Legend revised.

(5) Figure 6 double-positive reporter cells

Explain Tomato+GFP+ cells and their relationship to single-positive cells.

As described in our response to Reviewer 1, we now explain the kinetics of the mTmG reporter switch and interpret double-positive events as reporter-transition/persistence events rather than a separate lineage. This interpretation focuses the analysis on the biologically informative comparison— the different balance of FLT3-independent and FLT3-history labeling among TIMD4+ VCAM1+, TIMD4- VCAM1+, and monocyte-enriched populations.

Fate-mapping Results and Figure 6 Legend revised.

(6) CX3CR1 phenotype in Figures 6 and 7

The text describes CX3CR1 phenotype without showing the data.

We now describe Figures 6 and 7 using the markers actually displayed in those panels: TIMD4 and VCAM1. The relationship to CX3CR1 is established independently in Figure 2 and in the integrated scRNA-seq analysis. This separation makes the evidentiary chain clearer: Figure 2 links the surface-defined populations to CX3CR1 phenotype, Figure 6 compares their FLT3 reporter histories, and Figure 7 tests their CCR2 dependence.

Corresponding Results language and Figures 6-7 Legends revised.

(7) Figure 8 Csf1r-EGFP in thymocytes/DCs and “45.2”

Show Csf1r-EGFP expression in thymocytes and DCs; determine whether their reduction is indirect or direct; clarify 45.2.

The reviewer highlights why the MaFIA result should be interpreted at the Csf1r-expressingcompartment level. The reporter is demonstrably expressed by both TM populations and thymic monocytes, and AP20187 causes transgene-dependent TM depletion in FTOC. DC reduction may reflect direct transgene activity, dependence on the altered myeloid niche, or both; similarly, direct transgene expression was not measured in fetal DN thymocytes. We now make that cellular resolution explicit while emphasizing the developmental conclusion supported by the complete phenotype. We also clarify that CD45.2 denotes the congenic allele of the C57BL/6 control and not a distinct treatment or numerical value.

MaFIA Results, Discussion, and Figure 8 Legend revised.

(8) Figure 10 CD27 profiles

Show CD27 flow-cytometric profiles.

We revised the text and legend to make clear that Figure 10 reports quantified frequencies of CD27+ DN3 and DN4 cells. The CD27 measurement is interpreted in conjunction with, rather than in isolation from, the DN-stage data. Reduced CD27 among DN3 cells, reciprocal DN3 accumulation and DN4 loss, and reduced downstream DP output form a coherent sequence that localizes the developmental impairment to the b-selection-associated transition. We therefore use CD27 as a correlate of successful pre-TCR-associated progression rather than claiming direct biochemical measurement of pre-TCR signaling.

Final Results paragraph, Discussion, and Figure 10 Legend revised.

(9) Supplemental Figure 2 schemes

Improve the schemes and explain dLNGFR and AP20187.

The revised Figure Legend now explains each functional element. dLNGFR is the membrane-targeting low-affinity nerve growth factor receptor segment within the MaFIA fusion construct and is not used here as a lineage marker. AP20187 is a synthetic homodimerizer that binds the engineered FKBP domains, bringing the Fas intracellular domains together and initiating apoptosis in transgene-expressing cells. These definitions make the logic of the depletion system understandable without requiring familiarity with the original MaFIA construct.

Supplemental Figure 2 Legend revised.

Closing statement

The revised manuscript now presents the study in clear, evidence-matched terms. It identifies the advances in TM phenotypic organization, transcriptional specialization, developmental contribution, SpiC independence of total TM abundance, and CCR2 dependence of the TIMD4- VCAM1+ population. It also emphasizes the convergent evidence that Csf1r-expressing myeloid cells support progression through the DN3-to-DN4 checkpoint, while accurately defining the cellular resolution of the MaFIA experiment. We thank the editors and reviewers for helping us sharpen both the significance and the precision of these conclusions.
